# Osteopenia Due to Enhanced Cathepsin K Release by BK Channel Ablation in Osteoclasts

**DOI:** 10.1371/journal.pone.0021168

**Published:** 2011-06-14

**Authors:** Ulrike Sausbier, Christian Dullin, Jeannine Missbach-Guentner, Clement Kabagema, Katarina Flockerzie, Gerd Marten Kuscher, Walter Stuehmer, Winfried Neuhuber, Peter Ruth, Frauke Alves, Matthias Sausbier

**Affiliations:** 1 Department of Pharmacology and Toxicology, Institute of Pharmacy, University of Tübingen, Tübingen, Germany; 2 Department of Diagnostic Radiology, University Medical Center Göttingen, Göttingen, Germany; 3 Max-Planck-Institute for Experimental Medicine, Department of Molecular Biology of Neuronal Signals, Göttingen, Germany; 4 Institute for Anatomy, University of Erlangen-Nürnberg, Erlangen, Germany; 5 Department of Hematology and Oncology, University Medical Center Göttingen, Göttingen, Germany; Pennington Biomedical Research Center, United States of America

## Abstract

**Background:**

The process of bone resorption by osteoclasts is regulated by Cathepsin K, the lysosomal collagenase responsible for the degradation of the organic bone matrix during bone remodeling. Recently, Cathepsin K was regarded as a potential target for therapeutic intervention of osteoporosis. However, mechanisms leading to osteopenia, which is much more common in young female population and often appears to be the clinical pre-stage of idiopathic osteoporosis, still remain to be elucidated, and molecular targets need to be identified.

**Methodology/Principal Findings:**

We found, that in juvenile bone the large conductance, voltage and Ca^2+^-activated (BK) K^+^ channel, which links membrane depolarization and local increases in cytosolic calcium to hyperpolarizing K^+^ outward currents, is exclusively expressed in osteoclasts. In juvenile BK-deficient (BK^−/−^) female mice, plasma Cathepsin K levels were elevated two-fold when compared to wild-type littermates. This increase was linked to an osteopenic phenotype with reduced bone mineral density in long bones and enhanced porosity of trabecular meshwork in BK^−/−^ vertebrae as demonstrated by high-resolution flat-panel volume computed tomography and micro-CT. However, plasma levels of sRANKL, osteoprotegerin, estrogene, Ca^2+^ and triiodthyronine as well as osteoclastogenesis were not altered in BK^−/−^ females.

**Conclusion/Significance:**

Our findings suggest that the BK channel controls resorptive osteoclast activity by regulating Cathepsin K release. Targeted deletion of BK channel in mice resulted in an osteoclast-autonomous osteopenia, becoming apparent in juvenile females. Thus, the BK^−/−^ mouse-line represents a new model for juvenile osteopenia, and revealed the BK channel as putative new target for therapeutic controlling of osteoclast activity.

## Introduction

Osteopenia, which is discussed to be the clinical pre-stage of idiopathic osteoporosis, is characterized by a reduced bone mineral density and changes in bone micro-architecture leading to a higher risk for fragility fractures in juvenile females [1]. Its prevalence in the juvenile population of western industrialized countries is around 16 per cent and thus, osteopenia occurs more often in young pre-menopausal females than idiopathic osteoporosis, which has a prevalence of lower than one per cent in that group [2]. Progression of osteopenia often could result in an idiopathic osteoporosis of young females [3], characterized by fragility fractures of long bones and vertebrae, despite of normal hormone and vitamin levels and no other obvious reason for fragile bones. However, mechanisms leading to osteopenia still remain to be elucidated.

In the last decade it became apparent that potassium channels play a predominant role in idiopathic diseases such as LQT-syndrome, vasospastic angina pectoris, idiopathic epilepsy, episodic ataxia and paroxysmal movement disorders [4]–[15]. In human, 75 potassium channel types are known [16]. The large conductance, voltage and Ca^2+^-activated (BK) K^+^ channel is unique, since this channel links membrane depolarization and local increases in cytosolic calcium to hyperpolarizing K^+^ outward currents in many cell types [17]–[20].

Patch-clamp experiments using cultured chicken osteoclastic cells [21] as well as the osteoblastic human osteosarcoma cell lines MG-63 [22]–[24] and SaOS-2 [22], and the human osteogenic precursor cell line C1 [25] revealed the existence of a large conductance Ca^2+^-activated K^+^ channel in these cell types *in vitro*, which was assumed to be BK channel. However, its existence in these cell-lines is discussed controversially, since the measured single-channel conductance of this Ca^2+^-activated K^+^ channel-type does not always fit with the single-channel conductance of the BK channel. Furthermore, BK channel blockers were unable to block the large conductance hyperpolarizing K^+^ outward currents in some cell-lines [26]. Using a specific antibody against the carboxy-terminus of the pore-forming BK channel α-subunit [27] in femur and tibia slices, we found BK channels to be expressed only in osteoclasts, but not in osteoblasts or osteocytes. To study the functional relevance of osteoclast BK channel in bone *in vivo* and to evaluate its impact for juvenile osteopenia, we analyzed juvenile female wild-type (WT) and BK-deficient (BK^−/−^) mice [17] using *in vitro* and *in vivo* assays, flat-panel volumetric computed tomography (fpVCT) [28], [29] as well as high-resolution micro-CT (µCT).

## Methods

### Mice

BK channel-deficient mice (BK^−/−^) generated as previously described [17] were bred and maintained at the animal facility of the Institute of Pharmacy, Department Pharmacology & Toxicology, University of Tübingen, Germany. Either litter- or age-matched six to ten weeks old female wild type (WT) and BK^−/−^ mice with hybrid SV129/C57BL6 background (always F2 generation) were randomly assigned to the experimental procedures with respect to the German legislation on protection of animals. All *in vivo* experiments were approved by the local Ethics Committee for Animal Research of the University of Tübingen (approval-no.: 35/9185.81-2) and University Medical Center Göttingen (approval-no.: T24/08).

### Immunofluorescence of femur and tibia

Whole legs from 6 to 8 weeks old WT and BK^−/−^ mice were prepared, embedded in GSV1 embedding medium (Slee, Mainz, Germany) after removing the coat, snap-frozen in liquid nitrogen-cooled isopentane and then stored at −32°C. 12-µm-cryostat sections (Leica CM1900) were performed and mounted on Poly-L-Lysine (Sigma, München, Germany)-coated glass slides (Menzel, Braunschweig, Germany). After drying for one hour at room temperature (RT), slices were washed for 5 minutes with Tris-buffered-saline (TBS; 50 mM Tris, 150 mM NaCl; pH 7.4) and pre-incubated for one hour with 10% normal donkey serum (Dianova, Hamburg, Germany) in TBST (TBS containing 1% bovine serum albumin and 0.5% Triton X-100; pH 7.4). After rinsing with TBS for 5 minutes, slices were incubated over night at RT with anti-BKα_674–1115_ [27] in a dilution of 1∶1000 in TBST, washed three times for each 5 minutes with TBS, and incubated for one hour with a donkey anti rabbit-IgG conjugated with Alexa 555 (Molecular Probes [Göttingen, Germany]; 1∶1000 in TBST). After rinsing (3×5 minutes) with TBS, slices were incubated with DRAQ5 (Alexis Axxora [Lörrach, Germany]; 1∶1000 in TBST) for 15 minutes to visualize the cells' nuclei, washed again in TBS for three times 5 minutes each and were then cover-slipped in Kaiser's glycerine gelatine solution (Merck, Darmstadt, Germany). The slices were stored maximally for one day at 4°C under light protection until performing laser scanning microscopy.

For staining of Cathepsin K, cryo-sections were fixed for 5 minutes with −20°C-cold acetone at 4°C, rinsed three times for each 5 minutes with TBS and then pre-incubated with 10% normal donkey serum in TBST. After washing for 5 minutes with TBS, the primary antibodies anti-BKα_674–1115_ (1∶1000 in TBST; [27]) and anti-Cathepsin K (1∶50 in TBST; Santa Cruz, Heidelberg, Germany) were incubated over night at room temperature. After washing (3×5 minutes) with TBS, the sections were incubated for 1 hour with an Alexa555-conjugated donkey anti-rabbit IgG (Molecular Probes [Göttingen, Germany]; 1∶1000 in TBST) and an Alexa647-conjugated donkey anti-goat IgG (Molecular Probes [Göttingen, Germany]; 1∶1000 in TBST). The sections were washed with TBS (3×5 minutes) and cover-slipped in a 1∶1 mixture of TBS and glycerine (pH 8.6). Sections were stored under light protection at 4°C for maximally one day. Immunofluorescence was analyzed using a confocal laser scanning microscope (BioRad MRC1000) attached to a Nikon Diaphot 300 and equipped with a krypton-argon laser.

### DAB staining of femur and tibia

12-µm-cryostat sections (Leica CM1900) of long bones from 6 to 8 weeks old female WT and BK^−/−^ mice were performed and transferred on Poly-L-Lysine (Sigma-Aldrich, München, Germany)-coated glass slices (Menzel, Braunschweig, Germany). The sections were fixed with pre-chilled 2% paraformaldehyde for 5 minutes and washed with Tris-buffered saline (TBS; 50 mM Tris, 150 mM NaCl; pH 7.4). After blocking endogenous peroxidise activity with 0.3% H_2_O_2_/12.5% methanol in TBS for 15 minutes, sections were washed two times for each 5 minutes with TBS and pre-incubated for one hour with 5% normal goat serum (DAKO, Hamburg, Germany) in TBST (TBS containing 1% bovine serum albumin and 0.5% Triton X-100; pH 7.4). After rinsing with TBS, the slices were incubated overnight with anti-BK_(674–1115)_ (1∶250 in TBST; [27]), washed three times for each 5 minutes with TBS, and then tagged with horseradish-peroxidase-conjugated goat anti-rabbit IgG (1∶400 in TBST; DAKO, Hamburg, Germany) for 2 hours. After rinsing with TBS, immunoreactivity was visualized using the common DAB method. The sections were covered with Kaiser's glycerine gelatine solution (Merck, Darmstadt, Germany) and analyzed with a Nikon Eclipse microscope equipped with a digital camera.

### Tartrate-resistant acid phosphatase 5b staining

Mice were deeply anaesthetized with intraperitoneally applied ketamine/xylazine (100 mg/kg KG ketamine/10 mg/kg KG xylazine) and perfused via the left cardiac ventricle with 50 ml phosphate-buffered saline (PBS) followed by 100 ml 2% paraformaldehyde in PBS (2% PFA). After dissection of the coat the legs were prepared and post-fixed for 5 days in 2% PFA at 4°C. After washing with PBS for two consecutive days, the legs were decalcified in 0.4 M EDTA (pH 8.0) at room temperature for one month. Legs were washed with PBS for three days before being embedded in paraffin. Thereafter, longitudinal serial 10 µm sections were prepared on a SM2000 microtome (Leica, Wetzlar, Germany), mounted on Poly-L-Lysine (Sigma)-coated glass slides (Menzel, Braunschweig, Germany) and melted for 1 hour at 60°C. Serial sections were used for the staining of tartrate-resistant acid phosphatase (TRAP) using Naphthol AS-MX phosphate (Sigma, München, Germany) as substrate and Fast Red Violet LB Salt (Sigma, München, Germany) as coupler. After de-paraffinisation with xylol and rehydration via a descending line of ethanol, the sections were washed two times for 10 minutes each with 0.2 M sodium acetate buffer and incubated for 30 minutes in Fast Red Violet LB solution in a humidified chamber at 37°C. The Fast Red Violet LB solution contains 0.3 mg Fast Red Violet LB Salt (Sigma, München, Germany) per ml TRAP-buffer (17.6% 0.2 M sodium acetate buffer, 7.4% 0.2 M glacial acetic acid solution, 10% 0.3 M sodium tartrate buffer, 0.01% Naphthol AS-MX phosphate (Sigma, München, Germany) and 0.1% Triton X-100 (Sigma, München, Germany) in distilled water), which was tempered at 37°C before use. After incubation slices were rinsed with distilled water, counterstained with hematoxylin and covered in Kaiser's glycerine gelatine solution (Merck, Darmstadt, Germany). In five non-overlapping pictures of interest (POI) per slice, the number of nuclei per TRAP5b-positive cell (osteoclast) and the apparent number of osteoclasts within these POIs were evaluated by two independent researchers in a double-blinded manner using the light microscope Axiovert 200 M (Zeiss, Jena, Germany). The inter-slice interval was always 120 µm.

### Enzyme-linked immunoassays

Commercial available enzyme-linked immunoassays were used to analyze osteoprotegerin (OPG; Biozol, Eching, Germany) and sRANKL (soluble receptor activator of NF-κB ligand; Emelca Bioscience, Breda, Netherlands) as well as the osteoclast-derived cysteine protease Cathepsin K (Biozol, Eching, Germany) and the tartrate-resistant acid phosphatase 5b (TRAP5b; IDS, Frankfurt, Germany) in plasma from juvenile 6–8 weeks old WT and BK^−/−^ mice. The activity of Cathepsin K was determined by carboxyterminal telopeptides, the degradation product of collagen type I (IDS, Frankfurt, Germany). We also measured the levels of estradiol (IBL, Hamburg, Germany) and triiodothyronine (IBL, Hamburg, Germany). All experiments were carried out according to the manufacturer's protocol.

### Injection of recombinant murine sRANKL

2 µg recombinant murine sRANKL (R&D Systems, Wiesbaden, Germany; dissolved in physiologically buffered saline) was intraperitoneally injected into 5 WT and 5 BK^−/−^ female mice. Three hours later, the mice were sacrificed and CTX and TRAP5b were measured according to the manufacturer's protocol in plasma samples using a commercially available enzyme-linked immunoassay.

### Measurement of Calcium in mouse plasma

This was done by a commercial service of the central laboratory of the CRONA clinics (Tübingen, Germany) according to standard protocol.

### Flat-panel volume computed tomography (fpVCT)

fpVCT allows the *in vivo* acquisition of isotropic volume data sets with a resolution of about 150 to 200 µm for high-contrast structures such as bones. 6–8 weeks old WT and BK^−/−^ female mice were anaesthetized using a conventional isoflurane (1.5%–2%) inhalation regime, centered on the fpVCT gantry axis of rotation, and placed perpendicular to the *z*-axis of the system in order to scan the whole mouse with one rotation. The fpVCT (GE Global Research, Niskayuna, NY) consists of a modified circular CT gantry and two amorphous silicon flat-panel X-ray detectors (each with a size of 20.5×20.5 cm^2^) with a 1024×1024 matrix of 200×200-µm^2^ detector elements. The fpVCT works with a step-and-shoot acquisition mode. The standard *z*-coverage of one step was 4.21 cm. All data sets were acquired with the same protocol: 1000 projection images per rotation, 8-second rotation time, 360 used detector rows, 80 kVp, and 100 mA. A modified Feldkamp algorithm was used for image reconstruction resulting in high-resolution volume data sets (512×512 matrix), which were analyzed with voxtools 3.0.64 Advantage Workstation 4.2 (GE Healthcare, Buckinghamshire, UK). To suppress digitalization artifacts, the isotropic voxel size of reconstructed volume was about 100 µm and therefore less than the resolution of the system.

### High-resolution micro-CT (µCT)

To evaluate putative alterations in trabecular meshwork between the two genotypes, lumbar backbones from 6–10 weeks old WT and BK^−/−^ female mice were dissected and the 4^th^ and 5^th^ lumbar vertebrae were scanned with a high-resolution µCT eXplore Locus SP (GE Healthcare). Data sets were acquired with the following protocol: 1000 projection images, 360° scan technique, 5000 ms exposure time, 72 kV, 110 µA, and an isotropic resolution of 7.5 µm. Volume data sets with a 512×512 matrix were reconstructed using the proprietary software of the system and analyzed with a self-developed software implementing both the thinning algorithm from Lee and co-workers [30] and measurement tools for bone morphological parameters [31]. Thus, the trabecular meshwork could be evaluated in 3 dimensions (3D).

### Statistical analysis

All data are given as mean ± standard deviation (SD). Data's significance was evaluated using two-tailed Student's *t*-test, and values of *P*<0.05 were considered statistically significant.

## Results

### BK channel is expressed in native bone-degrading osteoclasts

**Figure 1:**
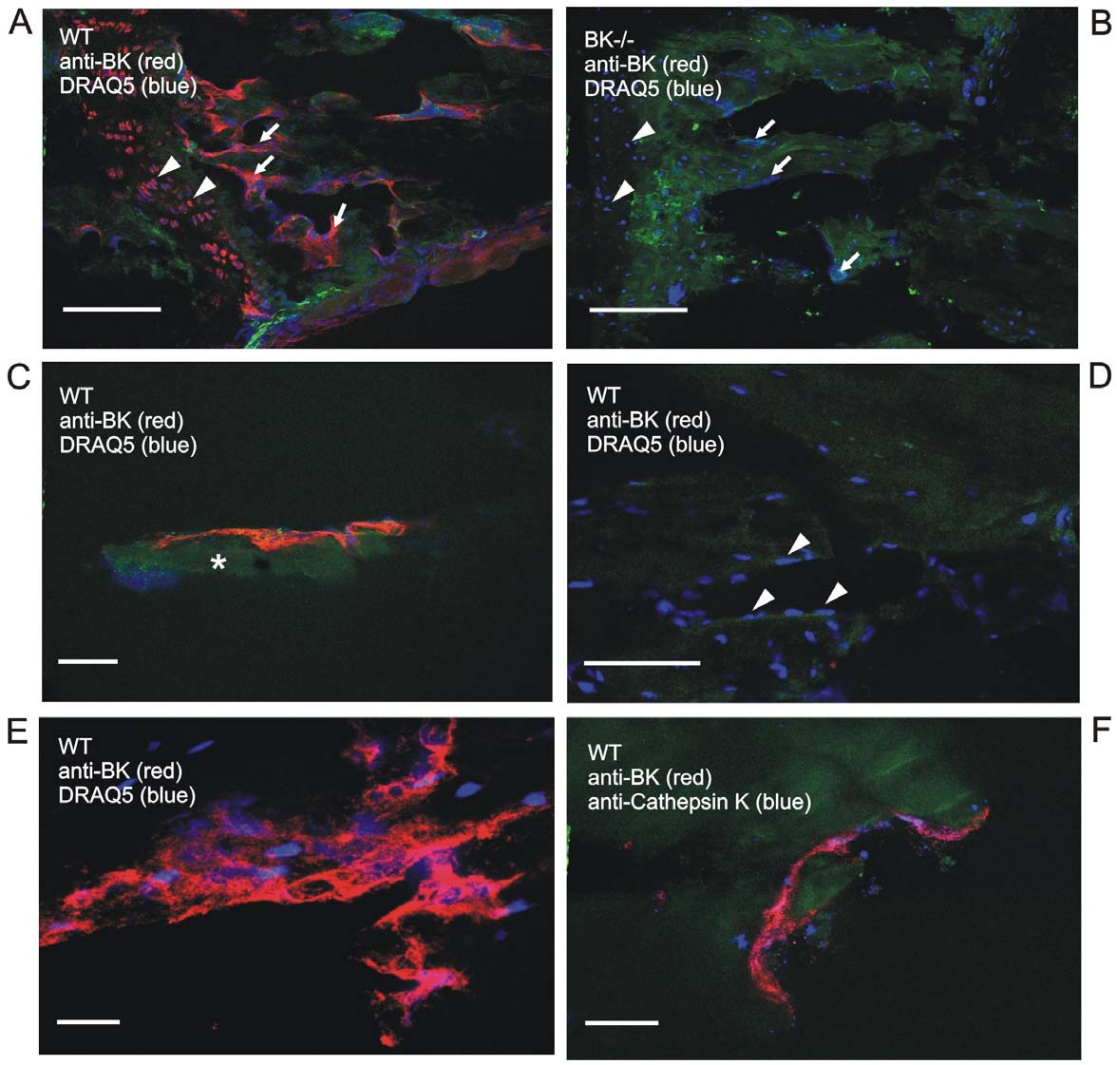
In native bone, BK channel is expressed in osteoclasts. (A) BK channel immunoreactivity (red) in chondrocytes (*triangles*) and large, multi-nucleated bone cells (*arrows*) within intact murine wild-type tibia, whereas BK^−/−^ tibia (B) lacks BK channel immunoreactivity. (C) A multi-nucleated bone cell (red) on a bone trabecula (*asterisk*) of tibia. (D) Single-nucleated osteoblasts (*triangles*) and osteocytes do not express BK channel in native tibia. Note, the red dot is an artificial anti-BK precipitate. (E) Magnification of a BK channel expressing multi-nucleated bone cell (red) in intact tibia. The nuclei were stained with DRAQ5 (blue). (F) Multi-nucleated bone cells expressing BK channel (red) were identified as osteoclasts by tagging the osteoclast-specific marker Cathepsin K (blue). Cathepsin K staining was detected in the Howship' lacuna as well as in cytosolic vesicles. Note, that native bone tissue shows green autofluorescence; scale bars: 100 µm (A, B), 50 µm (D), 25 µm (C, E, F).

To examine the expression profile of BK channel α-subunit in bone, we performed confocal laser scanning microscopy on native femur and tibia slices using the purified antibody anti-BKα_674–1115_ [17], [27]. In WT, immune reactivity was only detected in multi-nucleated bone cells, which were assumed to be osteoclasts, due to their size, their location within the bone and their number of nuclei (Figure 1). No BK channel staining was observed in BK^−/−^ bone (Figure 1B). To clearly identify these BK channel expressing multi-nucleated cells as osteoclasts, we performed immunohistochemistry for Cathepsin K, an osteoclast biomarker, using a specific antibody against this lysosomal cysteine protease (Figure 1F). Finally, immunofluorescence of native bone slices revealed that BK channel expression was only present in osteoclasts but not in osteoblasts nor in osteocytes (Figure 1A, D and also Figure S1).

### Increased Cathepsin K levels but normal bone-influencing endocrinology and osteoclastogenesis in BK^−/−^ mice

**Figure 2:**
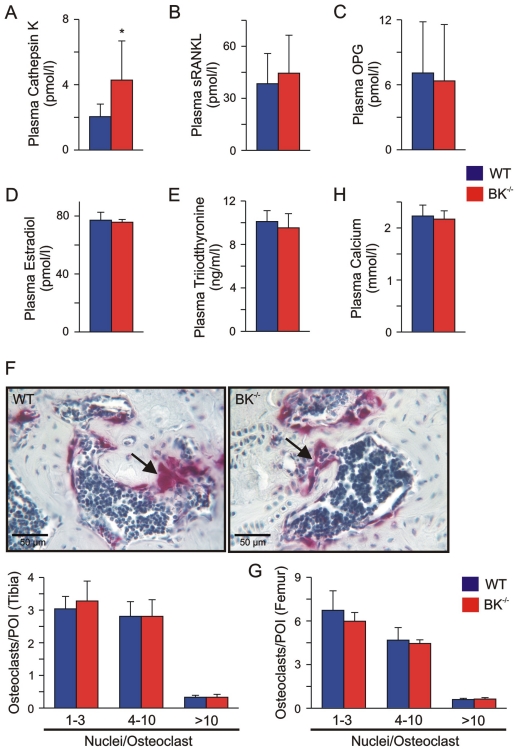
Enhanced plasma Cathepsin K, but normal endocrinology and osteoclastogenesis in juvenile BK^−/−^ mice. (A) Enhanced plasma Cathepsin K levels in juvenile BK^−/−^ mice. Statistics derived from 9 WT and 8 BK^−/−^ mice. (B, C) Osteoblast-derived factors influencing osteoclast activity were not altered in juvenile BK^−/−^ mice. Statistical summary of plasma sRANKL (B) and OPG (C) from WT and BK^−/−^ mice (n = 6–12 per genotype). (D, E) Endocrine factors regulating osteoclast activity were not altered in juvenile BK^−/−^ mice. Statistics of plasma estradiol (D) and plasma triiodothyronine (E) from WT (n = 12–17) and BK^−/−^ mice (n = 12–16). (F) *Upper:* Apparent number of osteoclasts in native BK^−/−^ proximal tibia differed not when compared to WT. Nuclei of TRAP5b-positive (purple) osteoclasts (arrow) were stained with hematoxylin (blue). *Lower:* Statistical summary of osteoclast quantification in tibia. (G) Statistics of osteoclast quantification in distal femur. In the cortical and cancellous compartment of the evaluated bones, TRAP5b-positive cells (osteoclasts) were counted by two independent persons in a double-blinded manner in five non-overlapping pictures of interest in each of 8–10 consecutive slices with an inter-slice-interval of 120 µm. The tibiae and femurs were derived from 4 WT and 4 BK^−/−^ mice. (H) Statistics of plasma Calcium in WT and BK^−/−^ mice (n = 9 per genotype). All data are means±SD; *P<0.05.

This large conductance, voltage and Ca^2+^-activated K^+^ channel links membrane depolarization and local increases in cytosolic calcium to hyperpolarizing K^+^ outward currents, thereby regulating Ca^2+^-dependent mechanisms such as activation in many cell-types. Osteoclasts expressing BK channel are important for bone matrix degradation and are thus, together with bone forming osteoblasts involved in bone remodeling. To answer the question, whether the BK channel is involved in regulation of osteoclast activity, we analyzed Cathepsin K as specific activity marker in plasma of juvenile WT and BK^−/−^ mice. Interestingly, plasma Cathepsin K levels in the mutants were about two-fold higher than in corresponding WT (BK^−/−^: 4.28±2.40 pmol/l, n = 8; WT: 2.04±0.77 pmol/l, n = 9) (Figure 2A), indicating that the osteoclast activity in BK^−/−^ mice was enhanced *in vivo*.

Acitvation of osteoclasts during bone remodeling is mediated by a variety of factors, which were derived from osteoblasts and T-lymphocytes but also from the endocrine system. Thus, the significantly increased plasma Cathepsin K levels in BK^−/−^ mice could be due to an enhanced activation of osteoclasts by bone cell-derived factors and/or by alterations of the endocrine system.

Soluble receptor activator of NF-κB ligand (sRANKL) is released from osteoblasts and their precursors as well as from T-lymphocytes, and stimulates the RANK-receptor on osteoclasts resulting in fusion of mono-nucleated osteoclasts and activation of multi-nucleated osteoclasts [32]–[35]. Via TRAF6-NFκB- and cFOS-signalling pathways RANKL induces nuclear factor of activated T-lymphocytes c1 (NFATc1), which in turn regulates osteoclastic gene expression such as of Cathepsin K and tartrate-resistant acid phosphatase 5b (TRAP5b) [32]–[34]. Analyzing plasma sRANKL by ELISA revealed no differences between both genotypes (WT: 38.45±17.40 pmol/l, n = 7; BK^−/−^: 44.49±21.90 pmol/l, n = 12), indicating that sRANKL secretion by osteoblasts and T-lymphocytes is not influenced by targeted deletion of BK channel (Figure 2B). Evaluating the plasma levels of the soluble decoy receptor osteoprotegerin (OPG), which is also secreted from osteoblasts and T-lymphocytes to prevent sRANKL interaction with its receptor, revealed again no differences between both genotypes (WT: 7.09±4.72 pmol/l; n = 8; BK^−/−^: 6.36±5.20 pmol/l, n = 6) (Figure 2C). These findings indicate that both osteoblast and T-lymphocyte activity is not influenced by genetic BK channel ablation. This is in agreement with the observation that BK channels are not expressed in native osteoblasts and in T-lymphocytes.

It is well established that osteoclast activity and subsequent bone remodeling is also modulated by the endocrine system of which estradiol, the thyroid hormone triiodothyronine and glucocorticoids are the prominent ones. Estradiol inhibits whereas triiodothyronine and glucocorticoids enhance osteoclast activity. Specific ELISAs revealed no alterations of plasma estradiol (WT: 77.1±5.5 pmol/l, n = 12; BK^−/−^: 75.7±1.9 pmol/l, n = 12) as well as plasma triiodothyronine (WT: 10.1±1.0 ng/ml, n = 17; BK^−/−^: 9.5±1.3 ng/ml, n = 16) between WT and mutant littermates (Figure 2D, E). Furthermore, in a previous study we could demonstrate that plasma glucocorticoid levels did not differ between the two genotypes [18]. Thus, the endocrine system influencing bone remodeling and subsequent osteoclast activity is not altered in BK^−/−^ mice compared to their WT littermates. These findings point to an osteoclast-autonomous phenotype being responsible for the increased plasma Cathepsin K levels in the mutant mice. However, the observed increased Cathepsin K levels in BK^−/−^ plasma might be due to an endogenous increased number of osteoclasts in BK^−/−^ bones. To evaluate this putative phenomenon, we stained serial paraffin sections from proximal tibia and distal femur for TRAP5b (Figure 2F), which is in bone an established specific and exclusive marker for osteoclasts. Counting TRAP5b-positive cells in five non-overlapping and randomly choosen pictures of interest in each of 8–10 consecutive slices (120 µm inter-slice-interval) revealed no differences in the apparent number of TRAP5b-positive cells between both genotypes (Figure 2F, G). To characterize putative alterations in osteoclast precursors, we evaluated the number of nuclei per TRAP5b-positve cell in the above mentioned sections (Figure 2F, G). Again, no significant changes were detected indicating that targeted deletion of BK channel in mice do not have an influence on osteoclastogenesis. Taken together, our results suggest an osteoclast-autonomous phenotype which is responsible for the increased Cathepsin K plasma levels in BK^−/−^ mice. However, the increased plasma Cathepsin K in BK^−/−^ mice had no influence on their plasma Ca^2+^ levels (WT: 2.23±0.27 mmol/l, n = 9; BK^−/−^: 2.17±0.16 mmol/l, n = 9) (Figure 2H), an indicator used in clinical diagnostics for rapid bone degradation as seen during bone metastasis.

### BK channel is involved in sRANKL-mediated Cathepsin K release *in vivo*

**Figure 3:**
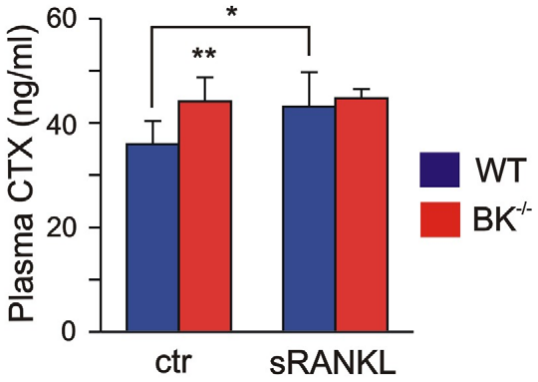
Enhanced released Cathepsin K is functional active in juvenile BK^−/−^ mice. Statistical summary of plasma level of characteristic collagen type I-degradation product CTX under basal conditions (ctr) and after intraperitoneal application of recombinant murine 2 µg sRANKL (n = 5–9 per genotype). Note, that CTX is a marker for functional active Cathepsin K. All data are means±SD; *P<0.05; **P<0.01.

The findings presented above raised the question whether the increased Cathepsin K level was functional operative in BK^−/−^ bones. The lysosomal cysteine protease Cathepsin K is important for the cleavage of collagen type I, the predominant organic component of bone matrix. The plasma level of the resulting C-terminal telopeptides (CTX) is described as clinical marker for bone resorption and correlates well with Cathepsin K activity in bone [36]. Determination of plasma CTX revealed an increase of this marker peptide (CTX in BK^−/−^: 44.1±4.6 ng/ml, n = 9; CTX in WT: 35.9±4.4 ng/ml, n = 8) (Figure 3), and thus verified the enhanced secreted Cathepsin K being functional operative in BK^−/−^ bones. This result supported the idea, that BK channel might be involved in the secretion of lysosomal cysteine protease Cathepsin K from osteoclasts, and favours the question whether *in vivo* collagen type I-degradation can be still more enhanced in the mutants in the presence of sRANKL. To test this, we applied murine sRANKL intraperitoneally and analyzed plasma CTX levels in WT and BK^−/−^ mice three hours later (Figure 3). In WT, we observed a significant increase in plasma CTX from 35.9±4.4 ng/ml (basal) up to 43.1±6.6 ng/ml (n = 5) after sRANKL application. In contrast to that, the plasma level of CTX from BK^−/−^ mice did not change significantly after sRANKL application (44.7±1.7 ng/ml, n = 5) when compared to basal conditions (44.1±4.6 ng/ml), indicating that osteoclast BK channel regulates the sRANKL-mediated secretion of Cathepsin K *in vivo*. The secretion of TRAP5b in a constant rate from osteoclasts was not influenced by BK channel ablation *in vivo* (Figure S2), suggesting a functional coupling between osteoclast BK channel activity and sRANKL-mediated Cathepsin K secretion.

### Flat-panel volumetric computed tomography (fpVCT) demonstrated reduced bone mineral density in juvenile BK^−/−^ mice

**Figure 4:**
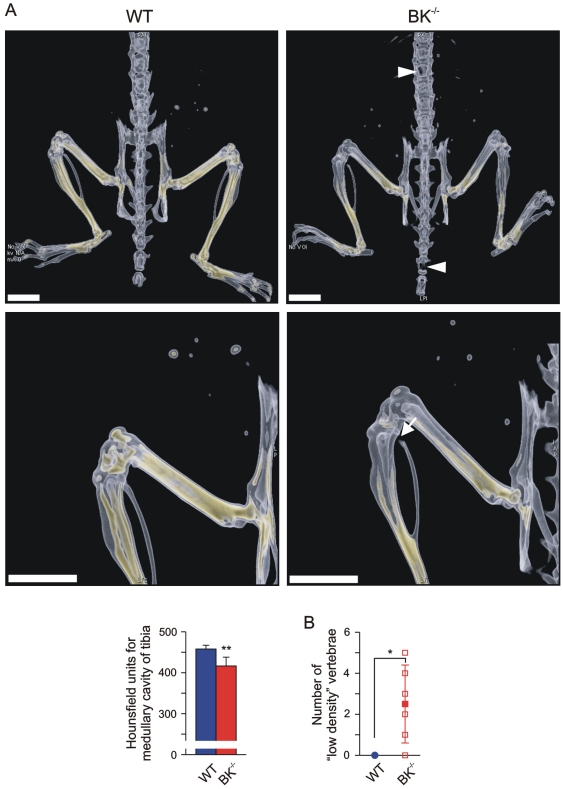
Reduced bone mineral density (BMD) in juvenile BK^−/−^ mice. (A) *Upper:* Representative dorsal views of volume rendering of legs, pelvic bone and backbone from a juvenile WT (*left*) and a BK^−/−^ mouse (*right*), and a magnification of their leg (*Middle*). BMD within the bones is reflected by a yellow-colour-scaling, whereas “yellow” encodes for high BMD values. Note the black gap (*arrow*) in BK^−/−^ fibula, and the “low-density” BK^−/−^ vertebrae and bones of the mouse tail (*triangle*), which was due to the very low BMD within that bone region; bar: 5 mm. *Lower:* Statistics of Hounsfield units in medullary cavity of tibia (n = 4–6 per genotype). (B) Statistical summary of “low-density” vertebrae apparent only in BK^−/−^ mice (n = 4–6 per genotype). All data are means±SD; *P<0.05; **P<0.01.

**Figure 5:**
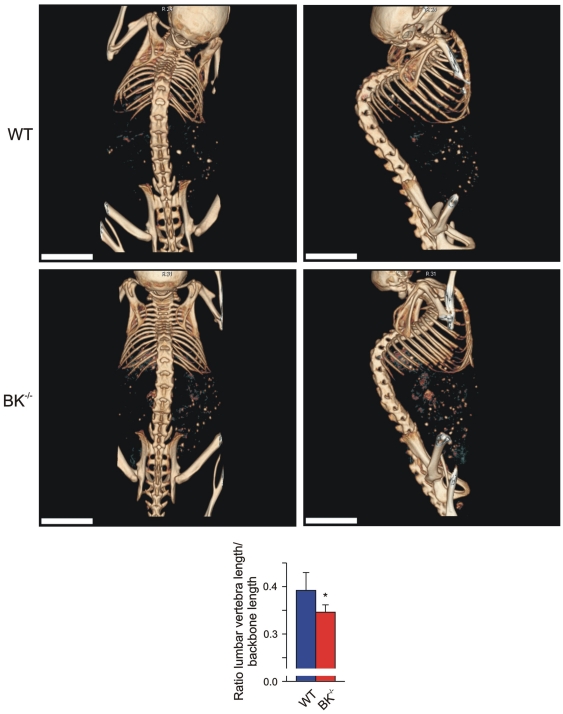
Osteopenic phenotype in juvenile BK^−/−^ mice. Representative, non-invasive imaging with fpVCT from total backbone in a dorsal (*left*) and lateral (*right*) view from a WT (*Upper*) and a BK^−/−^ mouse (*Middle*); bar: 5 mm. *Lower:* Statistical summary of ratio between length of 5 lumbar vertebrae and total length of backbone (n = 6 per genotype) obtained from non-invasive fpVCT imaging. All data are means±SD; *P<0.05; **P<0.01.

Our findings so far demonstrated an enhanced, functional operative Cathepsin K level due to the lack of BK channel in osteoclasts. This suggests putative alterations in bone mineral density (BMD) and/or trabecular meshwork formation in juvenile mice with targeted deletion of BK channel. To evaluate the *in vivo* consequences, we performed 3D high-resolution imaging of anaesthetized WT and BK^−/−^ mice by applying fpVCT. This technique enabled us to determine changes in BMD in living mice. According to osteoporosis diagnostic in patients we focussed on the analysis of murine femur and tibia. X-ray attenuation distributions were measured in Hounsfield-units (HU) and analyzed. BMD was calculated from HU by application of a linear function obtained from measurements of a bone density phantom: significant changes in HU between the two genotypes directly resemble different BMD. As shown in Figure 4, fpVCT imaging revealed in juvenile mutant mice a significant reduced BMD in the distal part of femur and in the proximal part of tibia. In-detail analysis of BMD in the medullary cavity of tibia revealed significantly reduced HU in the mutant (417±22 HU, n = 6) when compared to WT (458±9 HU, n = 4). Although fpVCT imaging suggested proximal fractures of BK^−/−^ fibulae (Figure 4), this was ruled out by macroscopic analysis after bone maceration: BK^−/−^ fibulae were all intact, but appeared fragile at the proximal side. At this side, the bone mineral density was so low that it could not be detected by fpVCT. The same phenomenon was observed in lumbar and thoracic BK^−/−^ vertebrae, where the bone mineral density detected by fpVCT was too low to have a measureable impact within the trabecular area (BK^−/−^: 2.5±1.9 vs. WT: 0±0) (Figure 4B). These “low density” vertebrae could be due to an increased BK^−/−^ osteoclast activity and subsequent enhanced Cathepsin K secretion, and may reflect an increased risk for fragility fractures. These findings favour an osteopenic phenotype in BK^−/−^ mice. Since the mechanostat mechanism in BK^−/−^ bones allows vertebrae to adapt to mechanical loading by differentiating their geometry, this could explain the finding, that the ratio between the length of 5 lumbar vertebrae and the length of total backbone was significantly reduced in BK^−/−^ (0.3460±0.0155 vs. WT: 0.3915±0.0377) mice (Figure 5). A putative dysregulation of the axial skeleton development in BK^−/−^ female mice could be ruled out in this context, since the ratio of tibia length and body length were not statistically significant between both genotypes (BK^−/−^: 0.186±0.005 vs. WT: 0.0187±0.002). Taken together, fpVCT discovered osteopenia in BK^−/−^ mice.

### High resolution micro-CT (µCT) revealed reduced trabecular density and meshwork in BK^−/−^ tibiae and vertebrae

**Figure 6:**
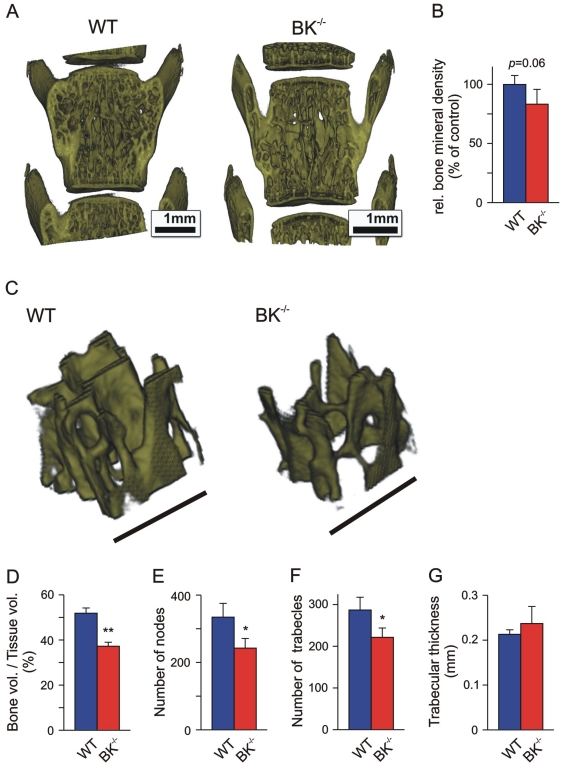
Increased porosity of BK^−/−^ vertebral trabecular meshwork. (A, B) Representative 3D reconstructions of the 4^th^ lumbar vertebra obtained by high-resolution µCT (A), and corresponding statistics of relative bone mineral density (B) from juvenile WT and BK^−/−^ mice (n = 4). (C–F) Representative reconstructions of a cubic region of interest (0.5×0.5×0.5 mm^3^) within the trabecular meshwork of the 4^th^ lumbar vertebra (C), and corresponding statistics of bone volume (BV)/tissue volume (TV)-ratio (D), number of nodes (E), trabecles (F) and trabecular thickness (G), evaluated in three cubic regions of interest (0.5×0.5×0.5 mm^3^). Bars: 1 mm (A), 500 µm (C); all data are means±SD; *P<0.05; **P<0.01.

fpVCT of backbone (see Figures 4 and 5) showed irregular and more compact morphology of BK^−/−^ lumbar vertebrae suggesting a reduced trabecular density and subsequent higher porosity. To evaluate and quantify putative alterations in trabecular micro-architecture in BK^−/−^ vertebrae we performed high-resolution µCT from 4 juvenile WT and BK^−/−^ mice each (Figure 6 and Table S1). A region of interest (2.0×1.5×1.5 mm^3^) within the trabecular compartment of the 4^th^ lumbar vertebra of each genotype was evaluated for relative bone mineral density (rBMD) (Figure 6A, B). In the analysed BK^−/−^ vertebrae the correlated rBMD was reduced (83.2±12.6%, n = 4) when compared to WT (99.9±7.5%, n = 4). Despite the missing significance (*P* = 0.06) in vertebral rBMD between the two genotypes, we found a significantly reduced bone volume (BV)/tissue volume (TV)-ratio in BK^−/−^ vertebrae (BK^−/−^: 37.22±1.79%, n = 4 vs. WT: 51.85±2.32%, n = 4) (Figure 6C, D). This clinical parameter was accompanied with a significantly reduced number of nodes (BK^−/−^: 242.5±28.5, n = 4 vs. WT: 334.4±41.0, n = 4) within the BK^−/−^ vertebral trabecular meshwork (Figure 6E) and a reduced number of trabecles *per se* (BK^−/−^: 221.4±22.4, n = 4 vs. WT: 287.0±30.6, n = 4) (Figure 6F), whereas the trabecular thickness were not significantly altered (BK^−/−^: 0.237±0.038 mm, n = 4 vs. WT: 0.213±0.010 mm, n = 4) (Figure 6G). Thus, these findings demonstrated a reduced trabecular meshwork density resulting in higher porosity of BK^−/−^ vertebrae, thereby reflecting a clinical hallmark of osteopenia. This finding was in principle confirmed in proximal BK^−/−^ tibiae showing significantly reduced bone volume of cortical and cancellous compartments (BK^−/−^: 1.659±0.278 mm^3^, n = 4 vs. WT: 2.024±0.320 mm^3^, n = 4) (Figure S3). The finding that distal femurs displayed a slightly reduced number of trabecular nodes (BK^−/−^: 599.8±183.8, n = 4 vs. WT: 720.2±213.2, n = 4) and trabecles (BK^−/−^: 517.2±151.4, n = 4 vs. WT: 627.3±175.6, n = 4), whereas the bone volume (BV)/tissue volume (TV)-ratio (BK^−/−^: 20.5±6.1%, n = 4 vs. WT: 18.0±4.0%, n = 4) and the trabecular thickness (BK^−/−^: 0.0267±0.0082 mm, n = 4 vs. WT: 0.0235±0.0066 mm, n = 4) were not statistically significant between both genotypes (Figure S4 and Table S1), are in-line with clinical observations in osteopenic models. However, in all three analyzed BK^−/−^ bone-types no fragility fractures could be detected by µCT, indicating that juvenile BK^−/−^ mice display an osteopenia rather than an osteoporotic phenotype.

## Discussion

Our data presented in this study establishes for the first time, that BK channels expressed in native osteoclasts control bone-degrading activity by regulating Cathepsin K release. Targeted deletion of BK channel in mice resulted in an osteoclast-autonomous osteopenia, being apparent in juvenile females displaying normal bone-influencing endocrinology and osteoclastogenesis, accompanied by increased plasma Cathepsin K levels, reduced bone mineral density and enhanced porosity of trabecular micro-architecture, but without obvious fragility fractures. Since juvenile osteopenia could result in an idiopathic osteoporotic phenotype, we focussed on analyzing juvenile female mice and provided new insights leading to juvenile osteopenia: loss of BK channel function in osteoclasts.

Immunohistochemistry of native bones using antibody anti-BKα_674–1115_ revealed a BK channel expression restricted to osteoclasts, whereas the BK channel was absent in osteoblasts and in osteocytes of intact bone. This suggests the BK channel as a specific biomarker to identify oestoclasts within bone. Our finding that osteoblasts do not express BK channels seems to conflict with electrophysiological studies using cultured osteoblasts [24], human osteosarcoma as well as immortalized osteoblast-precursor cell lines [22], [24], [25], [37]. In these cell lines, a large conductance, Ca^2+^-activated K^+^ outward current was recorded suggesting the existence of BK channels. However, this was also discussed controversially, because of the absence of the large conductance which is typical for BK channels [22], [26]. Another aspect contributing to the discrepancy of BK channel expression in cultured osteoblasts might be due to immortalization and the influence of serum factors under *in vitro* conditions. In fact, proteomic analysis of freshly isolated human osteoblasts from bone biopsies and of an immortalized osteosarcoma cell line revealed significant differences in their gene expression profiles [38]. Thus, cultured osteoblasts and cell-lines derived thereof differ from native osteoblasts of intact bone tissue in their BK channel expression.

An intact balance of osteoblast-derived sRANKL and OPG is essential for bone remodeling and subsequent for osteoclast activity. In bone, sRANKL exhibit a dual *in vivo* function: first, during osteoclastogenesis it activates the RANK-receptor on osteoclast precursors resulting in osteoclast differentiation and subsequent fusion to multi-nucleated osteoclasts [32]–[34], [39]. However, BK channel ablation in mice did not influence osteoclastogenesis as demonstrated by quantification of TRAP5b-positive osteoclasts and their nuclei. Second, sRANKL enhances multi-nucleated osteoclast activity promoting bone degradation. The osteoblast-derived decoy receptor for sRANKL, OPG, antagonizes these effects of sRANKL in bone remodeling. Thus, both sRANKL and OPG play a predominant role in triggering bone formation and degradation processes. An imbalance in this fine-tuned system promotes bone diseases like osteoporosis and osteopetrosis. This was previously demonstrated using mouse lines lacking either sRANKL or OPG. While mice deficient in sRANKL exhibited an osteopetrotic phenotype based on the lack of multi-nucleated osteoclasts due to an impaired osteoclastogenesis [40], the genetic deletion of OPG in mice resulted in an osteoporotic phenotype associated with an enhanced sRANKL-mediated activity of osteoclasts leading to a reduced bone mineral density, increased porosity and increased risk for fractures [41]. Taken together, enhanced Cathepsin K release from BK^−/−^ osteoclasts *in vivo* despite a normal cross-talk between bone-forming osteoblasts and bone-degrading osteoclasts as determined by analyzing sRANKL and OPG in plasma, and despite a normal endocrine system influencing osteoclast activity reflected by plasma levels of estradiol, triiodothyronine and glucocorticoids [18], pointed to an osteoclast-autonomous osteopenic phenotype.

How may the BK channel activity be related to the juvenile osteopenic phenotype? Osteoclast activity is regulated locally by hormones, growth factors, cell-matrix and cell-cell interactions, and mechanical stress [42]. The elevation and the decrease of [Ca^2+^]_i_ is very closely related with osteoclastic function [43]. Osteoclasts release the lysosomal acidic hydrolase Cathepsin K to digest the organic matrix of bone. The fusion of lysosomes with the ruffled border membrane is a prerequisite for bone resorption. Apparently, active osteoclasts use a Ca^2+^-dependent exocytosis machinery for Cathepsin K release [44]. For example, cytosolic Ca^2+^ increases when the osteoclasts adhere to the bone surface via their αvβ3 integrin receptors and start the digestion of the inorganic and organic matrix components. Subsequently, local Ca^2+^ concentrations increase in the resorbed area followed by a rise of [Ca^2+^]_i_ in osteoclasts. This rise in [Ca^2+^]_i_ is accompanied by a hyperpolarization of the plasma membrane, presumably to maintain the driving force for sustained Ca^2+^ entry [45]. As a result, resorbing activity gradually declines and the osteoclasts turn into non-resorbing and motile cells, which migrate and settle on a new surface area of the bone. The functional properties of the [Ca^2+^]_i_ rise may differ in resorbing versus non-resorbing osteoclasts and, in addition, the [Ca^2+^]_i_ elevation process in resorbing-state osteoclasts may not utilize the same Ca^2+^ entry pathway employed by non-resorbing osteoclasts. Several types of K^+^ and other ionic currents across the plasma membrane may help to maintain these functions of the osteoclast and the intracellular ion condition [46]. For an example, transport of H^+^ across the ruffled border leads to hyperpolarization of the osteoclast membrane, which would ultimately prevent further H^+^ efflux. The inwardly rectifying K^+^ conductance of a K_ir_ channel family member allows inward movement of K^+^ at hyperpolarized potentials, and has therefore been suggested to counteract the change in membrane potential arising from activity of the H^+^ pump [46]. On the other hand, the BK channel activity may be involved into the transition from the resorbing to the non-resorbing and motile state of osteoclasts, elicited by the hyperpolarization-induced increase of [Ca^2+^]_i_. This would imply that BK^−/−^ osteoclasts lacking the hyperpolarizing K^+^ outwards currents in response to increases of [Ca^2+^]_i_ will not easily enter into the non-resorbing and motile state at which bone-resorbing activity declines. Indeed, Ca^2+^-activated K^+^ currents have been suggested to be involved in the regulation of osteoclast movement and spreading on bone substrate [26]. Hyperpolarization of the cell membrane due to BK channel activity may serve as a positive feedback mechanism to increase Ca^2+^ influx through channels of the trp family [47]. After cessation of Ca^2+^ influx the positive feedback turns off, while the hyperpolarization due to BK channel activity can then reduce its own activity. Taken together, we suggest that the BK current contributes to the negative membrane potential of about −60 mV observed in non-resorbing/motile osteoclasts exhibiting pseudopodia but not exhibiting an actin ring [48]. The latter is being regarded as an indicator for the morphological and functional properties of resorbing osteoclasts. Hence, one may speculate that BK^−/−^ osteoclasts pause longer than wild-type in the resorbing state of their resorption cycle since their membrane potential may be similar to resorbing osteoclasts being approximately −10 to −20 mV. In fact, K channel activity and Ca^2+^ influx as a prerequisite for cell migration has also be shown for other cell types like mast cells [49].

Conclusively, we established the BK channel as regulator of osteoclast activity. Targeted deletion of BK channel revealed a juvenile osteopenia in mice accompanied with increased plasma Cathepsin K levels, reduced bone mineral density and increased porosity of trabecular meshworks of long bones and vertebrae, but without obvious fragility fractures. This mouse model may be useful to evaluate novel drug therapies for such a disorder to prevent putative progression from osteopenia to idiopathic osteoporosis in juvenile females.

## Supporting Information

Figure S1 **BK channel expression in WT, but not in BK^−/−^ osteoclasts.** Immunoreactivity in the epiphysis of tibia showed BK channel-expressing chondrocytes (*triangle*) and large, multi-nucleated osteoclasts (*arrow*). Other bone cell-types such as osteoblasts (area of osteoblasts is marked with an *asterisk*) and osteozyts displayed no staining for BK channel protein, as well as BK^−/−^ bone sections. Bars: 100 µm.
(TIF)

Figure S2 **TRAP5b secretion is not altered in BK^−/−^ osteoclasts.** Statistical summary of plasma level of TRAP5b under basal conditions (ctr) and after intraperitoneal application of recombinant murine 2 µg sRANKL (n = 4–12 per genotype and condition). Note, that TRAP5b is an osteoclast biomarker in immunohistochemistry and a correlate for the osteoclast number in bones. TRAP5b is released at a constant rate independent from osteoclast activity. All data are means±SD; *P<0.05; **P<0.01.
(TIF)

Figure S3 **Reduced bone volume in BK^−/−^ tibia assessed by µCT supported an osteopenic phenotype.** Statistics of the absolute bone volume comprising cortical and cancellous compartments in proximal tibia from juvenile WT and BK^−/−^ mice (n = 4 per genotpye). All data are means±SD; *P<0.05; **P<0.01.
(TIF)

Figure S4 **Bone parameters in BK^−/−^ femur assessed by µCT are not significantly altered.** Statistics of femoral bone volume (BV)/tissue volume (TV)-ratio (A), number of nodes (B), trabecles (C) and trabecular thickness (D), evaluated in three cubic regions of interest (0.5×0.5×0.5 mm^3^) in femurs from juvenile WT and BK^−/−^ mice (n = 4 per genotype). All data are means±SD; *P<0.05; **P<0.01.
(TIF)

Table S1 **Assessment of bone parameters in juvenile female mice using µCT.** Tb.S.: trabecular segmentation; Tb.N.: mean number of trabecular branching per trabecular node; Tb. Th.: trabecular thickness; all data are means±SD; *P<0.05; **P<0.01.
(DOC)
