# Supplementary figures and images for: Osteopenia Due to Enhanced Cathepsin K Release by BK Channel Ablation in Osteoclasts
Source: PLoS One. 2011 Jun 14;6(6):e21168. doi: 10.1371/journal.pone.0021168 (PMC3114853; doi:10.1371/journal.pone.0021168)

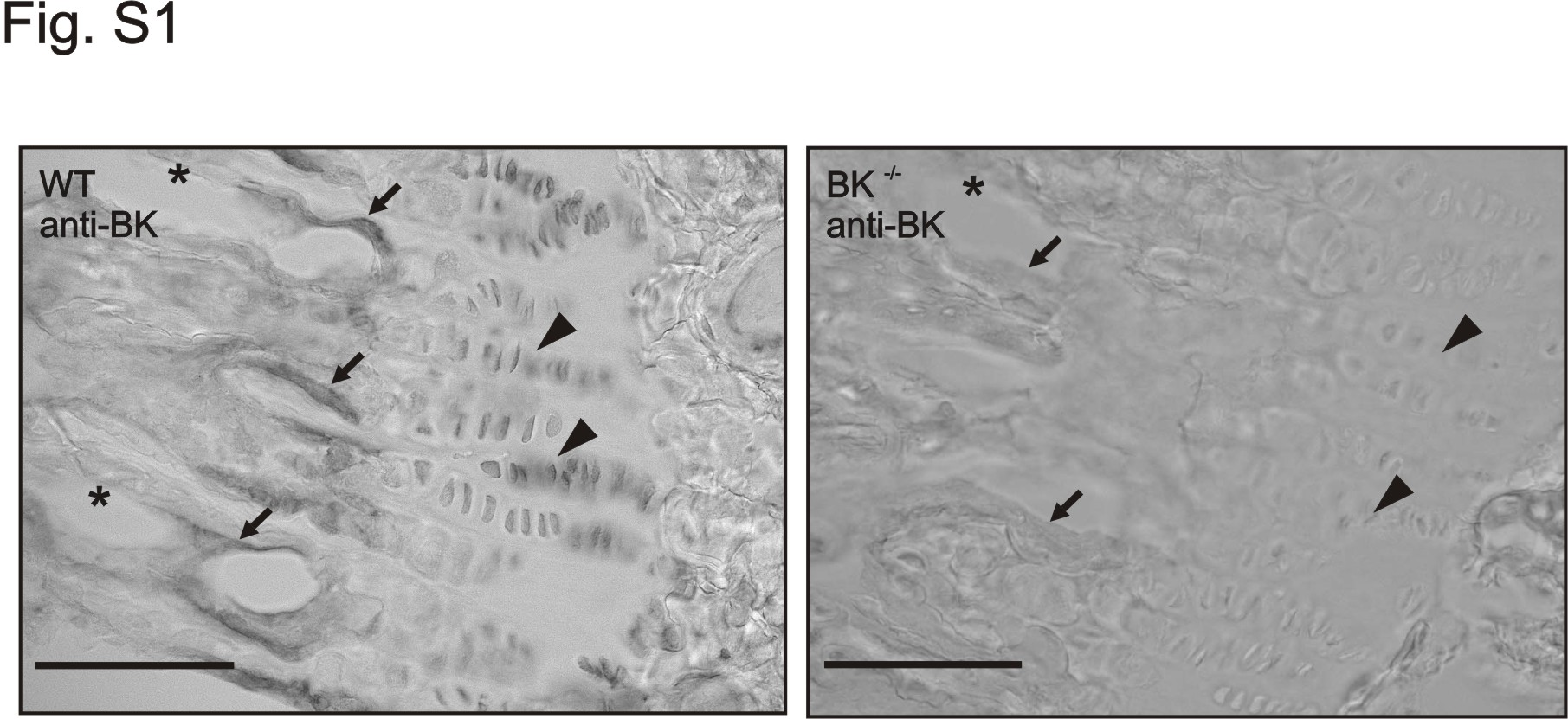

Supplement: Figure S1 — BK channel expression in WT, but not in BK−/− osteoclasts. Immunoreactivity in the epiphysis of tibia showed BK channel-expressing chondrocytes (triangle) and large, multi-nucleated osteoclasts (arrow). Other bone cell-types such as osteoblasts (area of osteoblasts is marked with an asterisk) and osteozyts displayed no staining for BK channel protein, as well as BK−/− bone sections. Bars: 100 µm. (TIF) [file pone.0021168.s001.tif]

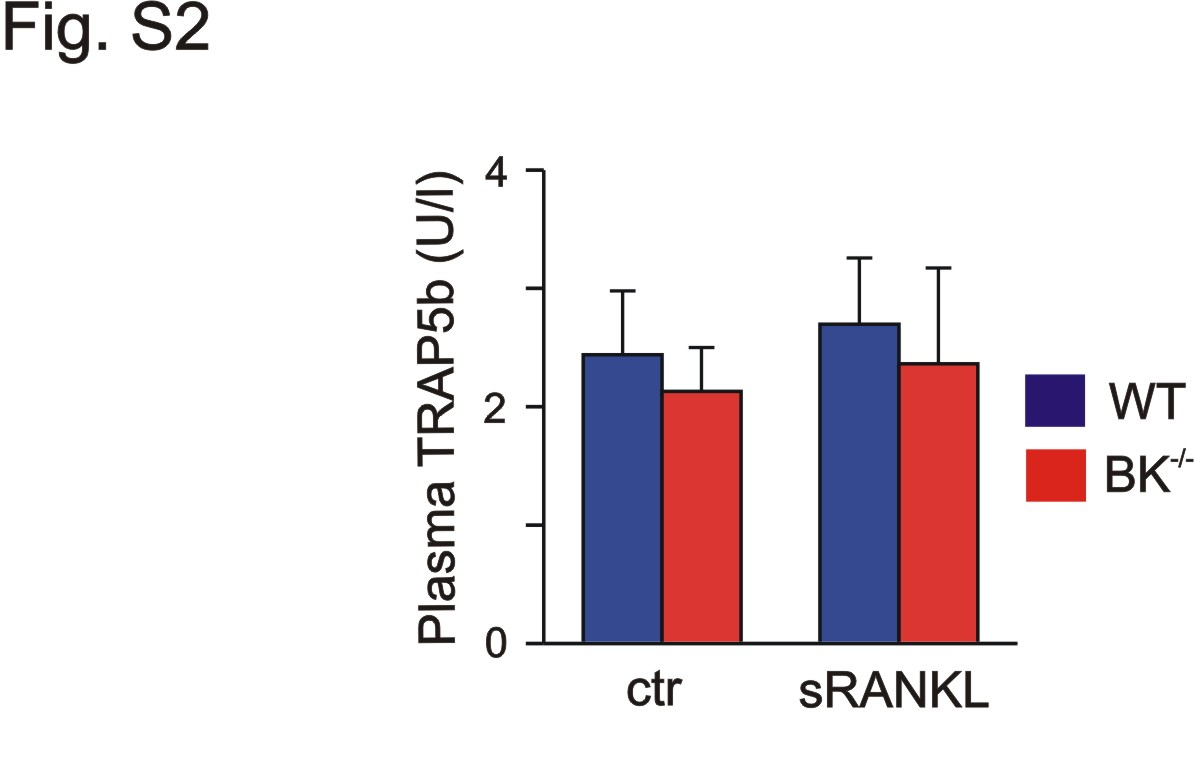

Supplement: Figure S2 — TRAP5b secretion is not altered in BK−/− osteoclasts. Statistical summary of plasma level of TRAP5b under basal conditions (ctr) and after intraperitoneal application of recombinant murine 2 µg sRANKL (n = 4–12 per genotype and condition). Note, that TRAP5b is an osteoclast biomarker in immunohistochemistry and a correlate for the osteoclast number in bones. TRAP5b is released at a constant rate independent from osteoclast activity. All data are means±SD; *P<0.05; **P<0.01. (TIF) [file pone.0021168.s002.tif]

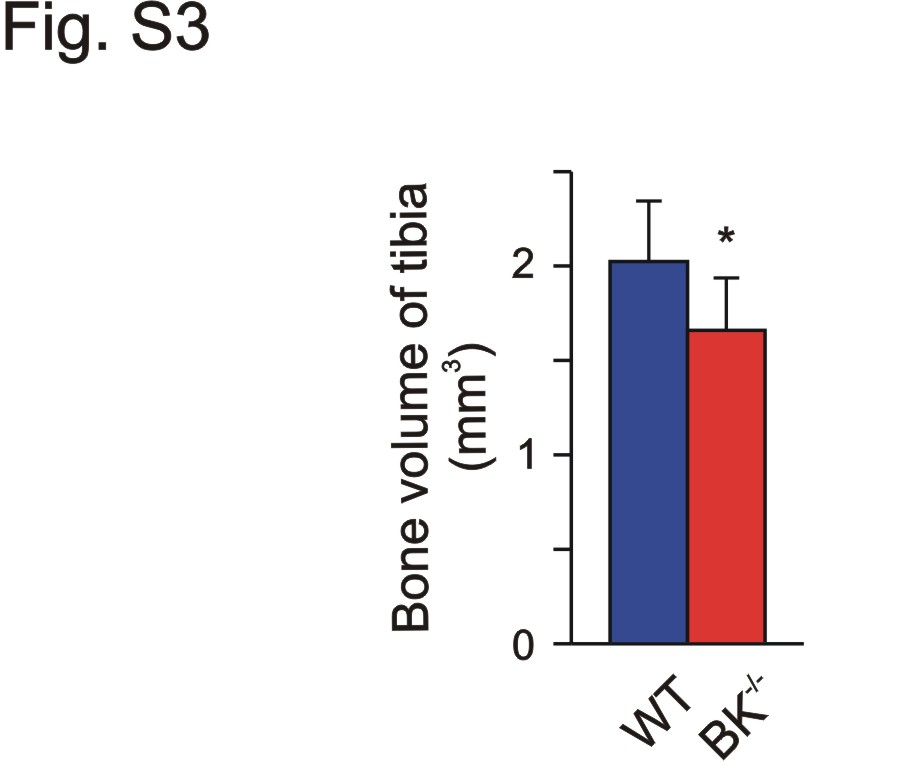

Supplement: Figure S3 — Reduced bone volume in BK−/− tibia assessed by µCT supported an osteopenic phenotype. Statistics of the absolute bone volume comprising cortical and cancellous compartments in proximal tibia from juvenile WT and BK−/− mice (n = 4 per genotpye). All data are means±SD; *P<0.05; **P<0.01. (TIF) [file pone.0021168.s003.tif]

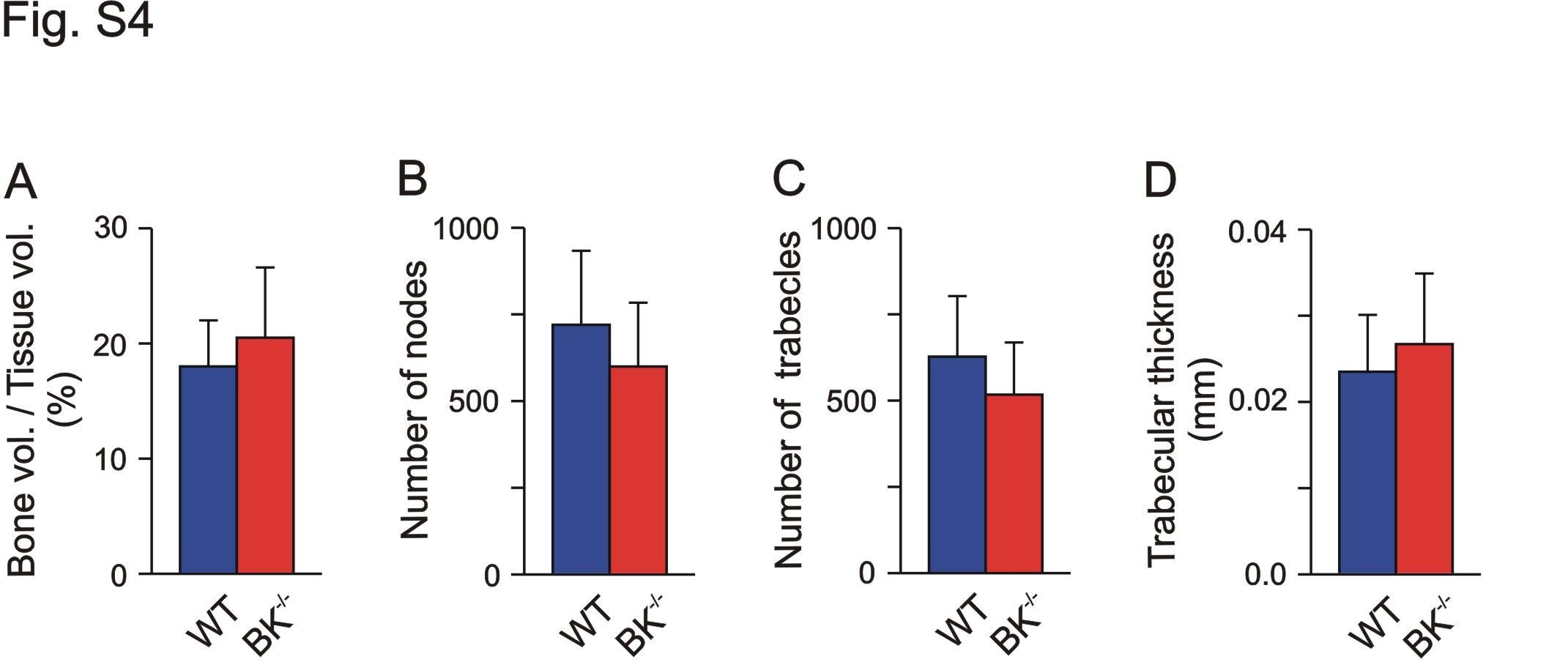

Supplement: Figure S4 — Bone parameters in BK−/− femur assessed by µCT are not significantly altered. Statistics of femoral bone volume (BV)/tissue volume (TV)-ratio (A), number of nodes (B), trabecles (C) and trabecular thickness (D), evaluated in three cubic regions of interest (0.5×0.5×0.5 mm3) in femurs from juvenile WT and BK−/− mice (n = 4 per genotype). All data are means±SD; *P<0.05; **P<0.01. (TIF) [file pone.0021168.s004.tif]
