# Supplementary material for: Osteopenia Due to Enhanced Cathepsin K Release by BK Channel Ablation in Osteoclasts
Source: PLoS One. 2011 Jun 14;6(6):e21168. doi: 10.1371/journal.pone.0021168 (PMC3114853; doi:10.1371/journal.pone.0021168)
Supplement: Table S1 — Assessment of bone parameters in juvenile female mice using µCT. Tb.S.: trabecular segmentation; Tb.N.: mean number of trabecular branching per trabecular node; Tb. Th.: trabecular thickness; all data are means±SD; *P<0.05; **P<0.01. (DOC) [file pone.0021168.s005.doc]

**Supporting Information Table**

**Table S1. Assessment of bone parameters in juvenile female mice using µCT.**

| **Lumbal vertebra** | | |
| --- | --- | --- |
| Parameter | **WT** (n=4) | **BK^-/-^** (n=4) |
| BV/TV (%) | 51.8±2.3 | 37.2±1.8 ** |
| Tb. S. (mm) | 0.054±0.004 | 0.054±0.005 |
| Tb. N. | 2.279±0.057 | 2.126±0.058 * |
| Tb. Th. (mm) | 0.213±0.010 | 0.237±0.038 |
| # nodes | 334.4±41.0 | 242.5±28.5 * |
| # trabecles | 287.0±30.6 | 221.4±22.4 * |
| **Femur** | | |
| Parameter | **WT** (n=4) | **BK^-/-^** (n=4) |
| BV/TV (%) | 18.0±4.0 | 20.5±6.1 |
| Tb. S. (mm) | 0.086±0.008 | 0.091±0.005 |
| Tb. N. | 2.331±0.115 | 2.420±0.114 |
| Tb. Th. (mm) | 0.0235±0.0066 | 0.0267±0.0083 |
| # nodes | 720.2±213.2 | 599.8±183.8 |
| # trabecles | 627.3±175.6 | 517.2±151.4 |
